# Green synthesis of enzyme/metal-organic framework composites with high stability in protein denaturing solvents

**DOI:** 10.1186/s40643-017-0154-8

**Published:** 2017-05-19

**Authors:** Xiaoling Wu, Cheng Yang, Jun Ge

**Affiliations:** Key Lab for Industrial Biocatalysis, Ministry of Education, Department of Chemical Engineering, Tsinghua University, Beijing, 100084 China

**Keywords:** Biocatalysis, Biomineralization, Immobilization

## Abstract

**Objectives:**

Enzyme/metal-organic framework composites with high stability in protein denaturing solvents were reported in this study.

**Results:**

Encapsulation of enzyme in metal-organic frameworks (MOFs) via co-precipitation process was realized, and the generality of the synthesis was validated by using cytochrome c, horseradish peroxidase, and *Candida antarctica* lipase B as model enzymes. The stability of encapsulated enzyme was greatly increased after immobilization on MOFs. Remarkably, when exposed to protein denaturing solvents including dimethyl sulfoxide, dimethyl formamide, methanol, and ethanol, the enzyme/MOF composites still preserved almost 100% of activity. In contrast, free enzymes retained no more than 20% of their original activities at the same condition. This study shows the extraordinary protecting effect of MOF shell on increasing enzyme stability at extremely harsh conditions.

**Conclusion:**

The enzyme immobilized in MOF exhibited enhanced thermal stability and high tolerance towards protein denaturing organic solvents.

**Electronic supplementary material:**

The online version of this article (doi:10.1186/s40643-017-0154-8) contains supplementary material, which is available to authorized users.

## Background

Enzymatic catalysis is one of the promising ways to achieve green industrial chemical processes. Immobilized enzyme is the most frequently used form of enzyme at industrial scale (Kirk et al. 2002; Bornscheuer et al. 2012). One of the important aims of enzyme immobilization is to achieve high enzyme stability at harsh conditions such as high temperature and organic solvents which often existe in industrial biocatalytic processes. Although immobilization may compromise the activity of enzyme, the greatly enhanced stability would enable the long-term and repeated use of enzyme and therefore a significantly reduced cost of enzyme (Liu et al. 2015; Liang et al. 2016a; Ji et al. 2016; Novak et al. 2015). Metal-organic frameworks (MOFs) have emerged as a new type of nanomaterials suitable for immobilization of enzyme (Feng et al. 2015; Lykourinou et al. 2011; Chen et al. 2012; Wen et al. 2016) and other biomolecules (Zhang et al. 2016; Li et al. 2016a, b). One major advantage of MOFs is the high chemical and structural stability of the nanoporous frameworks, which can offer a protective effect for encapsulated enzymes against adverse conditions while at the same time allow the transfer of small-molecule substrates/products.

Previous attempts in the preparation of enzyme-MOF composites have been majorly focused on the strategy of adsorbing protein molecules into pre-synthesized MOFs which have pore sizes similar to the dimensions of enzyme molecules (Feng et al. 2015; Lykourinou et al. 2011; Li et al. 2016a, b). Covalently linking enzyme molecules on the surface of MOF particles (Shi et al. 2016; Cao et al. 2016) was reported as another effective way for enzyme immobilization on MOFs, where the enzyme molecules were conjugated to the carboxylate groups on the MOFs. Co-precipitation or biomimetic mineralization (Lyu et al. 2014; Li et al. 2016a, b; Liang et al. 2015a, b, 2016b, c; Wu et al. 2015a, b; He et al. 2016; Shieh et al. 2015), a self-assembly process to encapsulate bioactive molecules within the protective exteriors, has been successfully introduced to the synthesis of enzyme-MOF composites. Typically, in a biomimetic mineralization process, protein molecules in aqueous solution first binds with metal ions or organic ligands to form nanoscaled aggregates which induce the nucleation of MOF crystals. Then, the MOF crystals grow as the shell of protein aggregates to encapsulate enzyme inside. The enzyme-MOF composites have showed the great potential of increasing enzyme stability at high temperature (Liang et al. 2015a). However, the stability of enzyme-MOF composites in organic solvents, especially in highly polar organic solvents such as dimethyl sulfoxide (DMSO), dimethyl formamide (DMF), methanol, and ethanol has not been well investigated.

Here, we report a biomimetic mineralization route to synthesize enzyme-MOF composites, using cytochrome c (Cyt c), horseradish peroxidase (HRP), and *Candida antarctica* lipase B (CALB) as model enzymes. After being encapsulated in MOFs, enzymes can retain almost all their activities even incubated in protein denaturing solvents including DMSO, DMF, methanol, and ethanol. In contrast, free enzymes lost almost all their activities at the same condition.

## Experimental section

### Materials

Horseradish peroxidase (HRP) (reagent grade), *Candida antarctica* lipase (CALB), 2-methylimidazole, phosphate buffer saline (1×), 1,2,3-trihydroxybenzene (THB, ACS reagent), and 4-nitrophenyl butyrate (p-NPB) were purchased from Sigma-Aldrich. Zinc nitrate hexahydrate (99.998%) and hydrogen peroxide (29–32% wt) were purchased from Alfa Aesar. Cytochrome c from horse heart was purchased from Biodee Corporation. All the other reagents are of analytic grade.

### Synthesis of the enzyme/ZIF-8 composite

HRP, CALB, Cyt c water solution (5 mg/mL, 4 mL), and Zn(NO_3_)_2_ water solution (92.5 mg/mL, 4 mL) were added into 2-methylimidazole water solution (4.1 g dissolved in 40 mL water), followed by stirring for 30 min at room temperature. The mixture turned milky almost instantly after mixing. After aged at room temperature for 12 h, the product was collected by centrifugation at 6000 rpm for 10 min, washed with DI water for three times, and used for further characterization.

### Transmission electron microscopy (TEM)

Methanol solution of ZIF-8 and enzyme/ZIF-8 composite was placed on the carbon-coated support grid and dried at room temperature for TEM measurement and energy dispersive X-ray spectrometry (EDS) analysis on a JEOL JEM-2010 high-resolution TEM with an accelerating voltage of 120 kV.

### XRD analysis of ZIF-8, enzyme/ZIF-8 composite

Powder X-ray diffraction (XRD) patterns were conducted using a Bruker D8 Advance X-Ray diffractometer with a Cu Kα anode (*λ* = 0.15406 nm) at 40 kV and 40 mA.

### Thermogravimetric analysis of ZIF-8, enzyme/ZIF-8 composites

Samples were heated from room temperature to 600 °C at a rate of 10 °C/min under air atmosphere on a TA Instruments TGA 2050 Thermogravimetric Analyzer.

### Activity assay of enzyme/ZIF-8 composite and its free counterpart

The activity of HRP was determined by measuring the rate of decomposition of hydrogen peroxide with THB, which can be converted to a yellowish product, purpurogallin, detectable at 420 nm. In a typical experiment, HRP/ZIF-8 was added to a solution containing H_2_O_2_ (9 μM) and THB (16 mM) in phosphate buffer saline. After reaction for 10 min, the solution was centrifuged for 2 min at 12,000 rpm. And the absorbance of the supernatant was recorded at 420 nm on a UV–Vis spectrophotometer. The activity of free HRP was measured using the same procedure.

For the enzymatic activity determination of Cyt c/ZIF-8 and free Cyt c, similar procedure was followed by shortening the reaction time to 3 min.

For the activity assay of CALB/ZIF-8, p-NPB was first dissolved in acetone and then diluted with phosphate buffer (50 mM, pH 7.0) containing 1.25% (w/v) Triton X-100. The composite of CALB/ZIF-8 was added to the phosphate buffer containing 4-nitrophenyl butyrate (p-NPB) (0.5 mM) to initiate the reaction. After reaction for 3 min, the solution was centrifuged at 12,000 rpm for 2 min. The absorbance of the supernatant was determined at 400 nm by using a UV/Vis spectrophotometer.

### Enzyme stability in denaturing organic solvents

Enzyme/ZIF-8 composites and corresponding free enzyme powders were incubated in water, dimethyl sulfoxide (DMSO), dimethyl formamide (DMF) at 80 °C and in boiling methanol and ethanol for 1 h. Tiny amount of the enzyme solution was taken out and diluted to appropriate concentration and subjected to the above enzymatic assays. The relative activity of enzyme/ZIF-8 was calculated as the ratio of the activity of treated enzyme/ZIF-8 exposing to high temperature and organic solvents after required immersion time and activity of the untreated enzyme/ZIF-8 (Eq. 1). The activity of the untreated enzyme/ZIF-8 was set as 100%. The relative activity of free enzyme was calculated in the same way.

1 $${\text{Relative Activity}}= \frac{{{{{\text{Activity}}\;{\text{of}}\;{\text{Enzyme}}} /{{\text{ZIF-}}8\;{\text{treated}}}}}}{{{{{\text{Activity}}\;{\text{of}}\;{\text{Enzyme}}} / {{\text{ZIF-}}8\;{\text{untreated}}}}}} \times 100\%.$$

## Results and discussion

**Fig. 1:**
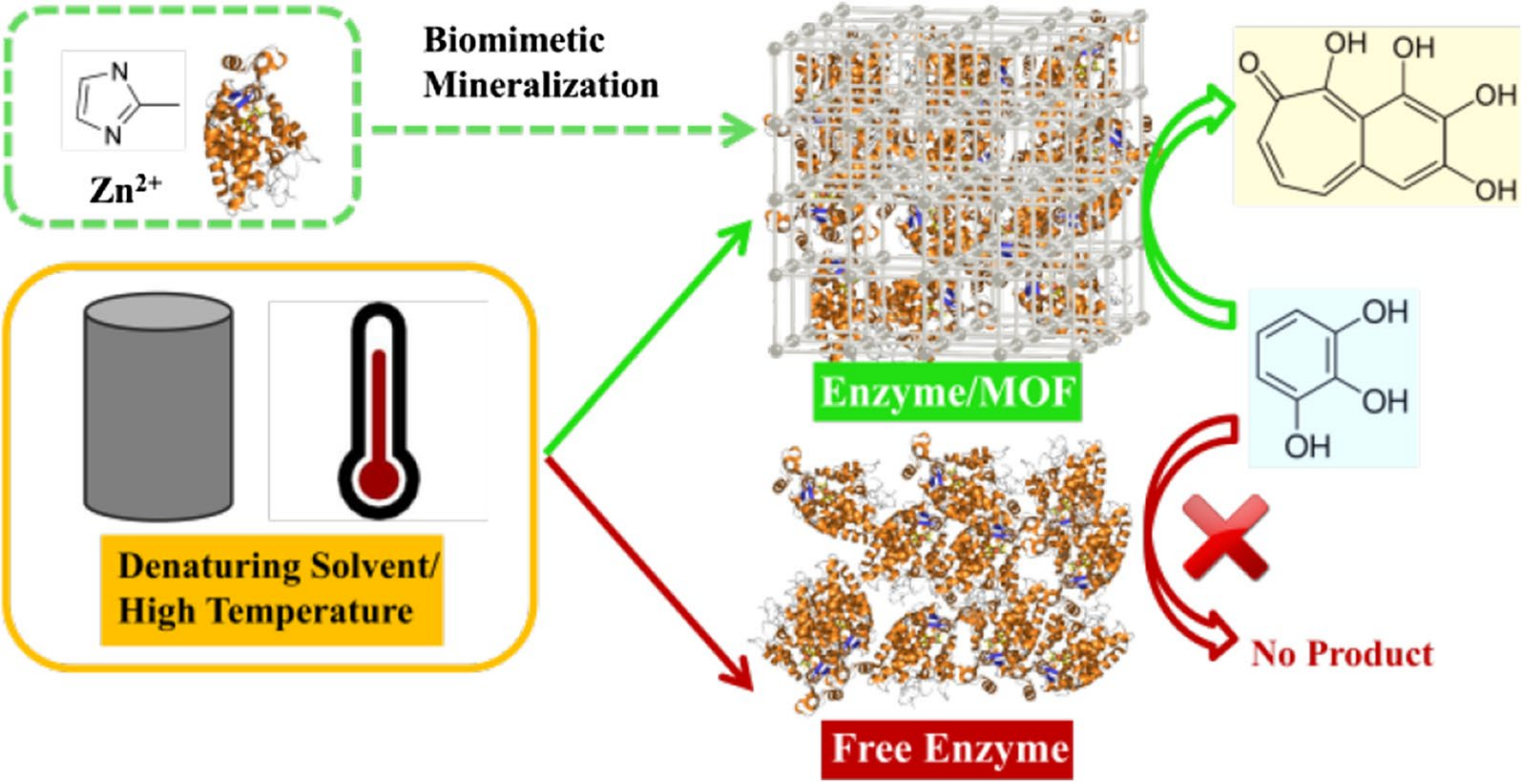
Scheme of the green synthesis of enzyme-MOF composites exhibiting tolerance for denaturing solvents and heat

As shown in Fig. 1, in this study, using cytochrome c (Cyt c), horseradish peroxidase (HRP), and *Candida antarctica* lipase B (CALB) as model enzymes, the synthesis of enzyme-MOF composites was carried out by mixing 4 mL of enzyme solution (5 mg/mL), 4 mL of zinc nitrate water solution (310 mM), and 40 mL of 2-methylimidazole water solution (1.25 M). The synthesis was conducted in water solution at room temperature for 30 min. Protein induced the nucleation of zeolitic imidazolate frameworks-8 (ZIF-8), and ZIF-8 framework grew around enzyme molecule providing a protective shell. After aging at room temperature for 12 h, the product was collected by centrifugation at 6000 rpm for 10 min, followed by removing the adsorbed protein by three cycles of washing and centrifugation.

**Fig. 2:**
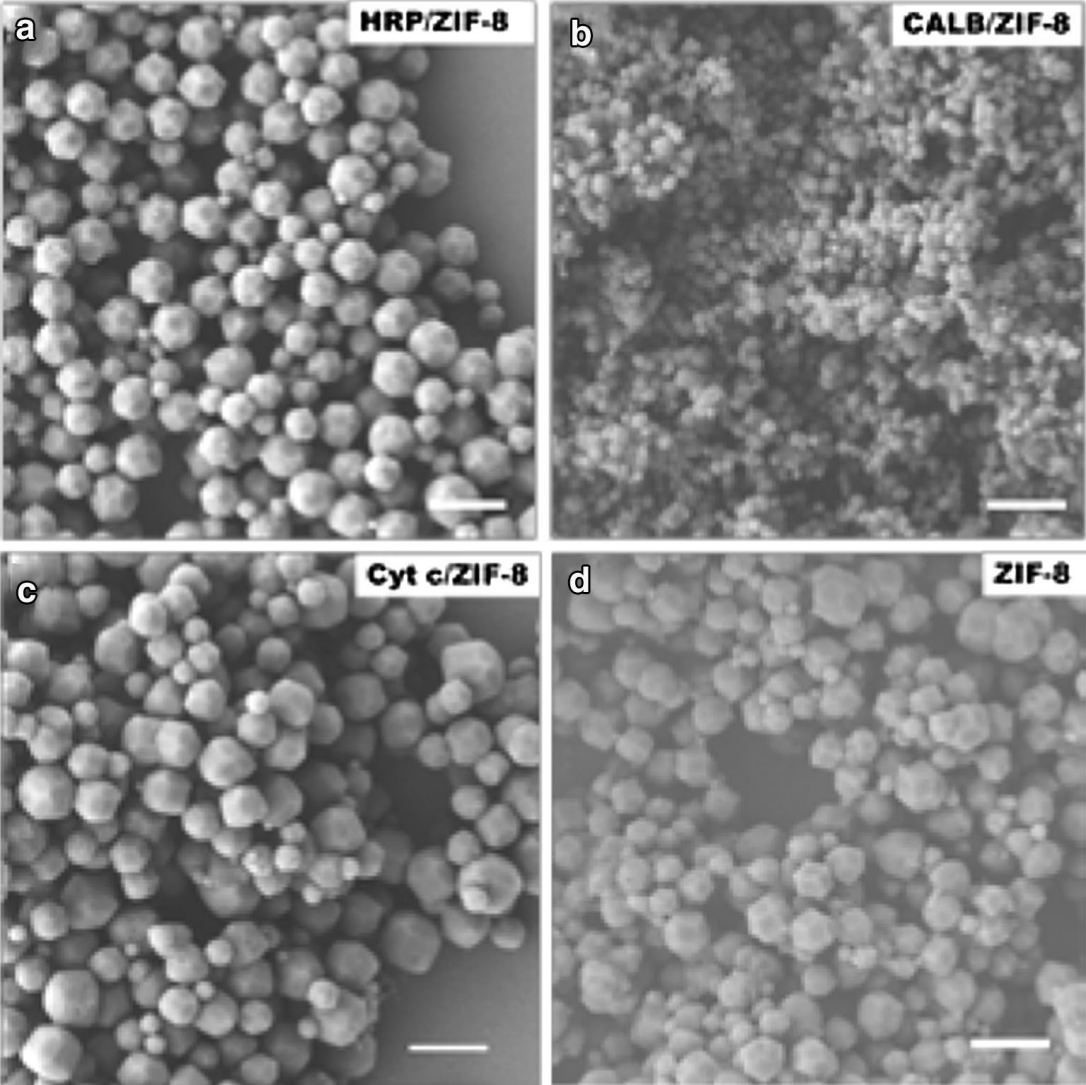
SEM images of **a** HRP/ZIF-8, **b** CALB/ZIF-8, **c** Cyt c/ZIF-8, and **d** ZIF-8. *Scale bars* 1 μm

**Fig. 3:**
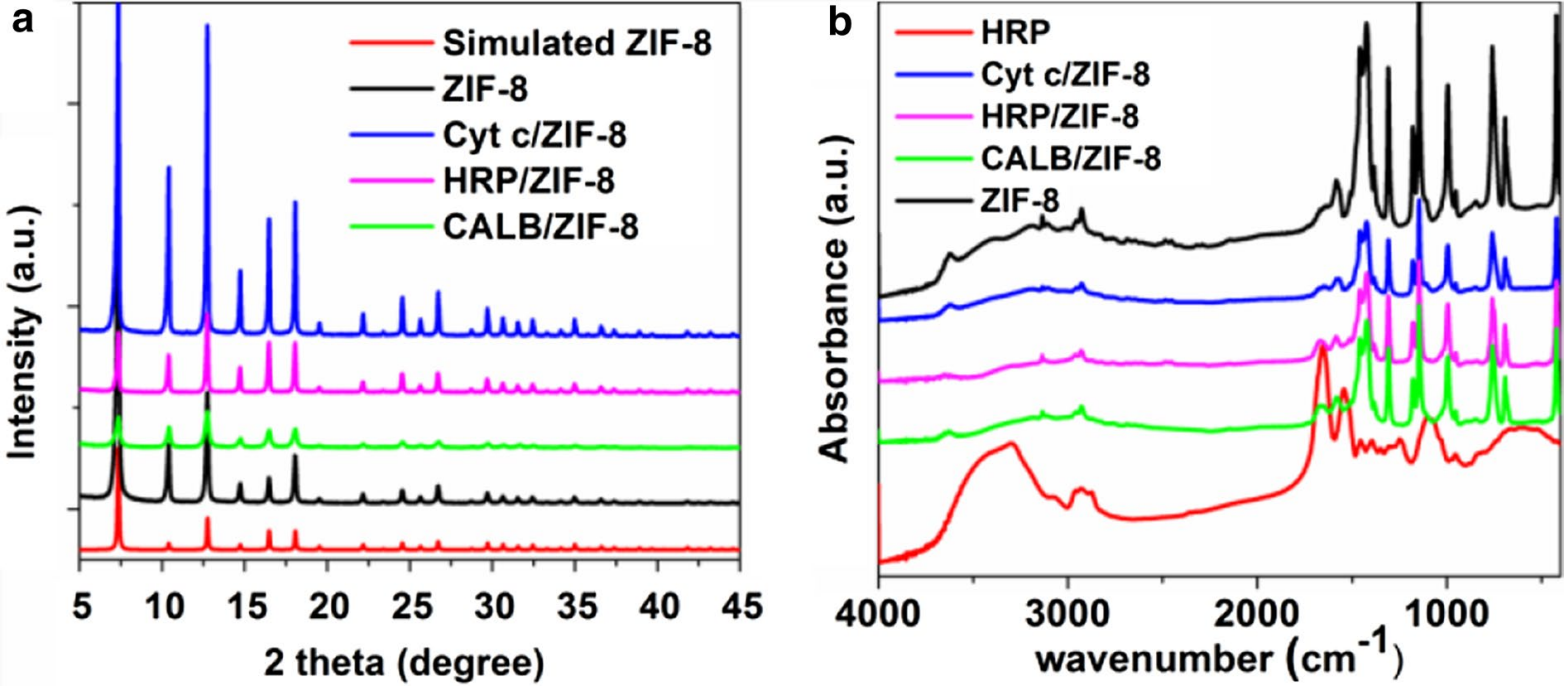
**a** XRD patterns of ZIF-8, Cyt c/ZIF-8, HRP/ZIF-8, CALB/ZIF-8, and simulated ZIF-8. **b** FT-IR spectrum of ZIF-8, Cyt c/ZIF-8, HRP/ZIF-8, CALB/ZIF-8, and HRP

As shown in Fig. 2 and Additional file 1: Figures S1, S2, the scanning electron microscope (SEM) images and transmission electron microscope (TEM) images of HRP, CALB, Cyt c/ZIF-8 nanocomposites showed similar morphologies to that of pure ZIF-8, with sizes ranging from ~100 to ~800 nm. It seemed that the size of the enzyme/MOF composites was widely distributed due to the rapid nucleation and diverse growth of the crystals. The X-ray diffraction (XRD) patterns of the enzyme/ZIF-8 composites agreed well with the patterns of simulated ZIF-8 and pure ZIF-8 (Fig. 3a), which verified that the incorporation of enzyme did not affect the crystallinity of ZIF-8.

The Fourier transform infrared spectroscopy (FTIR) spectra (Fig. 3b, 1640–1660 cm^−1^ corresponding to amide I band mainly from C=O stretching mode) proved the presence of protein in the composites. The thermal gravity analysis (TGA) in air also confirmed the presence of protein in the composites (Additional file 1: Figure S3). The loading percentages of protein in the composites interpreted from TGA curves were 10, 5, and 10% for HRP, CALB, and Cyt c, respectively, which is consistent with the result of size exclusion chromatography.

To further confirm that enzymes were embedded in ZIF-8 instead of adsorbing on the external surface, control experiments were carried out by physically mixing the as-prepared pure ZIF-8 crystals with enzyme solutions to form the enzyme-adsorbed ZIF-8 samples. After the same washing step, both the enzyme-embedded ZIF-8 (enzyme/ZIF-8) and enzyme-adsorbed ZIF-8 (enzyme@ZIF-8) samples were digested by acetic acid, followed by sodium dodecyl sulfate-polyacrylamide gel electrophoresis (SDS-PAGE, Additional file 1) performed on an analytic polyacrylamide (12%) gel. As shown in Additional file 1: Figure S4, in SDS-PAGE, protein bands corresponding to respective molecular weights of enzymes (12 kD for Cyt c, 38 kD for CALB, and 44 kD for HRP) appeared on the gel for both free enzymes and enzyme/ZIF-8 samples (lane 1 and lane 2, respectively). In contrast, no obvious bands were observed for all three types of enzyme@ZIF-8 samples (lane 3). The result demonstrated that the embedded enzymes in ZIF-8 scaffolds cannot be removed by simple washing step but only can be released out from ZIF-8 scaffolds once the ZIF-8 was digested by acetic acid. In contrast, the enzyme molecules adsorbed on the external surface of ZIF-8 crystals were completely removed in the washing step.

The specific activities of the synthesized enzyme/ZIF-8 composites were determined at the same protein concentration as native enzymes (Additional file 1). HRP/ZIF-8 and CALB/ZIF-8 retained 12 and 5% of activity compared to native enzymes. Please note that the small aperture size of ZIF-8 is 3.5 Å, indicating the transport of substrate such as 4-nitrophenyl butyrate through the small aperture must be very difficult. However, previous studies showed that enzyme was embedded in ZIF-8 crystals in the form of protein aggregates with size around 20–30 nm (Lyu et al. 2014). The relatively large cavities localizing protein aggregates were presented in both the surface and inside of enzyme-MOF composites, and these cavities were partially connected (Lyu et al. 2014). In addition, in the biomimetic mineralization, structural defects of crystals usually formed due to the presence of protein in the crystallization process. These cavities and structural defects in composites could possibly serve as the major routes for transferring substrates. Moreover, studies also demonstrated that the pores of MOFs have a “breathing” effect which could enlarge the aperture size to some extent to allow the transferring of molecules (Serre et al. 2002). All these evidence and results provided the possible mechanism for substrate transportation inside the enzyme-MOF composites. The low activity was possibly due to the activity loss during the immobilization process and/or the hindered mass transfer caused by the ZIF-8 framework, which was commonly observed in most cases of immobilized enzymes (Kim et al. 2008; Brady and Jordaan 2009; Hanefeld et al. 2009). However, Cyt c embedded in ZIF-8 showed a sixfold increase in activity compared to free Cyt c in solution. The control experiment confirmed that the pure ZIF-8 had no catalytic activity towards the substrate. The high activity of Cyt c in ZIF-8 was possibly due to the increased substrate affinity resulted from a conformational change of Cyt c to expose its heme group (Ono and Goto 2006) in the appropriate microenvironment created by ZIF-8. Moreover, the interaction between the embedded Cyt c and Zn^2+^ in ZIF-8 crystals also might contribute to the enhancement of the catalytic activity of Cyt c (Lyu et al. 2014; Ge et al. 2012). The mechanism of the increased activity of Cyt c in MOF has been investigated elsewhere (Lyu et al. 2014).

**Fig. 4:**
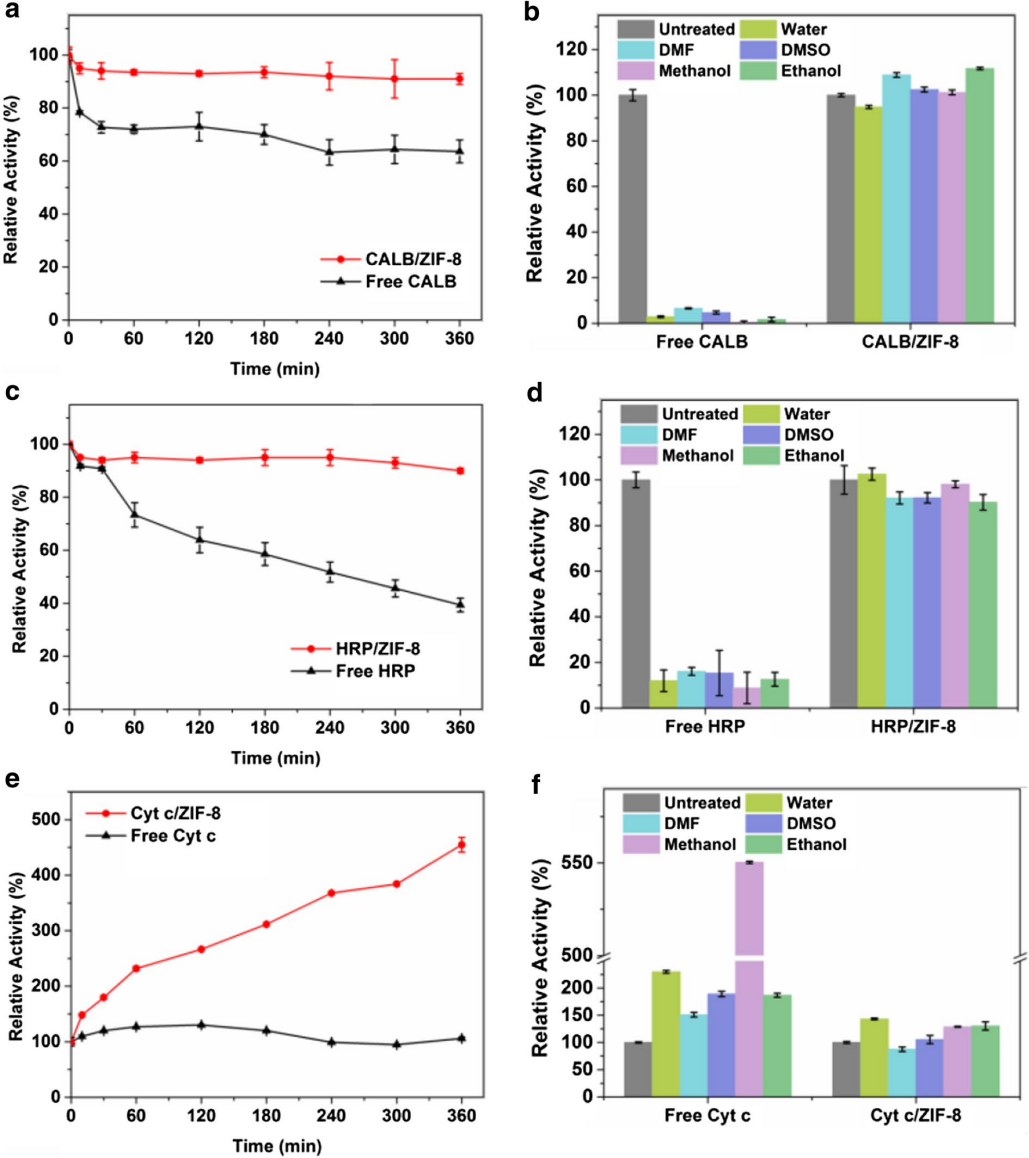
**a**, **c**, **e** Stability of CALB/ZIF-8, HRP/ZIF-8, Cyt c/ZIF-8 incubated in water for 6 h at 40, 50, and 70 °C, respectively. **b**, **d**, **f** Comparison of stabilities of enzymes and corresponding enzyme-MOF composites in water, DMF, DMSO at 80 °C and in boiling methanol and boiling ethanol. Enzymes and corresponding enzyme-MOF composited were incubated in the above solvents for 1 h and taken out for enzymatic assays

The thermal stability of enzymes embedded in ZIF-8 was evaluated by comparing the residual activity of free enzymes and enzyme/ZIF-8 composites after incubating in phosphate buffer saline solution at high temperatures (70 °C for Cyt c, 50 °C for HRP, 40 °C for CALB). As shown in Fig. 4, after 6-h incubation all enzyme/ZIF-8 composites maintained over 90% of their original activities while free enzymes lost 60 and 30% of their original activities for HRP and CALB, respectively. The greatly enhanced stability of enzyme/ZIF-8 composites can be attributed to the rigid structure and the confinement effect of MOF scaffolds that repress protein conformational transition or unfolding at high temperatures. As an exceptional case, free Cyt c itself had a good stability in water solution at 70 °C, with almost no loss of activity during the 6 h incubation (Fig. 4e). For Cyt c/ZIF-8, an increased activity up to 450% was observed during the incubation at 70 °C, which might be caused by the increased accessibility of heme group in Cyt c at high temperature which has been discussed previously (Lee et al. 2005; Pace and Hermans 1975; Fujita et al. 2007). Long-term storage stability is important for immobilized enzyme. As shown in Additional file 1: Figure S5, the HRP/ZIF-8 retained half of its original activity even after 4-day incubation at room temperature. We attributed the retention of the activity to the protecting effect from the rigid structure of ZIF-8 for maintaining the conformation of encapsulated enzyme. We evaluated the recyclability of HRP/ZIF-8. As shown in Additional file 1: Figure S6, the activity of HRP/ZIF-8 was decreased by 50% at the third cycle of reusing. The loss of activity might be ascribed to the difficulty of recovering all the nano-sized composites during the centrifugation process in the recovery.

To examine the tolerance of enzyme/ZIF-8 composite to polar and hydrophilic solvents at high temperature, the enzyme/ZIF-8 composites were incubated in water, DMF, DMSO at 80 °C and in boiling methanol, boiling ethanol for 1 h. As shown in Fig. 4, by using 4-nitrophenyl butyrate and 1,2,3-trihydroxybenzene as substrate, native CALB was almost fully deactivated in all the above conditions and native HRP retained no more than 20% of its original activity. In contrast, at the same condition, the activity of CALB/ZIF-8, HRP/ZIF-8, and Cyt c/ZIF-8 remained almost unchanged, demonstrating the unprecedented protecting effect of the framework. In the case of free Cyt c, an enhancement of 50–450% was observed after incubation in these solvents. The exceptional activity can again be ascribed to the exposure of heme in Cyt c under the condition where the active sites of Cyt c became more accessible to substrates, which facilitates the catalysis process (Ono and Goto 2006). When Cyt c was encapsulated in ZIF-8, the protecting effect of the framework resisted the protein configuration change in denaturing organic solvents, which resulted in almost unchanged of activity (Fig. 4f).

These organic solvents including DMF, DMSO, methanol, and ethanol are recognized as very effective denaturing reagents for most proteins, because they can seriously destroy the structure of protein due to their high polarity and solubilization capability. It has been investigated that protein molecules in such denaturing solvents quickly lost the tertiary and secondary structures, presenting as unfolded or partially unfolded configurations (Desai and Klibanov 1995; Xu et al. 1997). The deactivation of enzyme induced by denaturing solvents is irreversible. Because of the serious denaturing effect, almost no free enzyme is reported to retain reasonable activity after incubating in these pure organic solvents (DMF, DMSO, methanol, and ethanol). Protein engineering (including direct evolution and random or site-directed mutagenesis) which is a very effective tool for improving enzyme stability under various circumstances also found its difficulty in solving this problem. Protein engineering has only been proven to be effective for increasing enzyme stability in aqueous-denaturing solvent mixtures. For example, random mutagenesis resulted in more stable subtilisin, which can only increase its stability in 60% DMF (Chen and Arnold 1991). Therefore, the retention of activity in these anhydrous denaturing organic solvents was still far from enough since these solvents such as DMSO and DMF usually served as commonly used solvents for the synthesis of polysaccharides (Ferreira et al. 2002), peptide (Nilsson and Mosbach 1984), etc. (Bordusa 2002). Recent studies on enzyme-MOF composites demonstrated the capability of improving enzyme catalytic performance in aqueous solutions (Feng et al. 2015; Lykourinou et al. 2011; Chen et al. 2012). In this study, we showed that the confinement of enzyme in ZIF-8 framework provided an effective way to increase enzyme stability in denaturing organic solvents. The confinement of ZIF-8 can prevent the encapsulated protein molecules from conformational changes and therefore retain the protein structure integrity, while the free enzyme was directly exposed to the denaturing organic solvents leading to serious change of protein configuration and loss of activity.

## Conclusions

Enzyme/ZIF-8 composites were prepared by the biomimetic mineralization process. The one-step synthesis was carried out in aqueous solution at room temperature within 30 min. The structural rigidity and confinement of MOF scaffolds greatly enhanced the thermal stability of embedded enzymes. In protein denaturing organic solvents including DMF, DMSO and boiling methanol and ethanol, free enzymes were almost fully deactivated, while enzyme/ZIF-8 composites retained all its original activity after incubation in these solvents for 1 h. This study demonstrated a green chemistry way of preparing immobilized enzymes based on MOFs to achieve high enzyme stability at harsh conditions.

## Additional file

**Additional file 1.** Additional information.

## Abbreviations

MOF: metal-organic framework
Cyt c: cytochrome c
HRP: horseradish peroxidase
CALB: *Candida antarctica* lipase B
DMSO: dimethyl sulfoxide
DMF: dimethyl formamide
ZIF-8: zeolitic imidazolate frameworks-8
SEM: scanning electron microscope
TEM: transmission electron microscope
XRD: X-ray diffraction
FTIR: Fourier transform infrared spectroscopy
TGA: thermal gravity analysis
SDS-PAGE: sodium dodecyl sulfate-polyacrylamide gel electrophoresis
