# Supplementary material for: Green synthesis of enzyme/metal-organic framework composites with high stability in protein denaturing solvents
Source: Bioresour Bioprocess. 2017 May 19;4(1):24. doi: 10.1186/s40643-017-0154-8 (PMC5438438; doi:10.1186/s40643-017-0154-8)
Supplement: Supplementary file 1 — Additional file 1. Additional information. [file 40643_2017_154_MOESM1_ESM.docx]

**Supporting information for**

**Green synthesis of enzyme/metal-organic framework composites with high stability in protein denaturing solvents**

**Xiaoling Wu, ^a^ Cheng Yang, ^a^ Jun Ge *^,a^**

*^a^* Key Lab for Industrial Biocatalysis, Ministry of Education, Department of Chemical Engineering, Tsinghua University, Beijing 100084, China

**To whom correspondence should be addressed.**

E-mail: [junge@mail.tsinghua.edu.cn](mailto:junge@mail.tsinghua.edu.cn)

**Experimental Section**

**Materials**

Horseradish peroxidase (HRP) (reagent grade), Candida antarctic lipase (CALB), 2-methylimidazole, phosphate buffer saline (1x), 1,2,3-trihydroxybenzene (THB, ACS reagent) and 4-nitrophenyl butyrate were purchased from Sigma-Aldrich. Zinc nitrate hexahydrate (99.998%) and hydrogen peroxide (29-32% wt) were purchased from Alfa Aesar. Cytochrome c from horse heart was purchased from Biodee Corporation. All the other reagents are of analytic grade.

**Synthesis of the enzyme/ZIF-8 composite.**

HRP, CALB, Cyt c water solution (5 mg/mL, 4 mL) and Zn(NO_3_)_2_ water solution (92.5 mg/mL, 4 mL) were added into 2-methylimidazole water solution (4.1 g dissolved in 40 mL water), followed by stirring for 30 min at room temperature. The mixture turned milky almost instantly after mixing. After aged at room temperature for 12 h, the product was collected by centrifugation at 6 000 rpm for 10 min, washed with DI water for three times and used for further characterization.

**Transmission electron microscopy (TEM)**

Methanol solution of ZIF-8 and enzyme/ZIF-8 composite was placed on the carbon-coated support grid and dried at room temperature for TEM measurement and energy dispersive X-ray spectrometry (EDS) analysis on a JEOL JEM-2010 high-resolution TEM with an accelerating voltage of 120 kV.

**XRD analysis of ZIF-8, Enzyme/ZIF-8 composite**

Powder X-ray diffraction (XRD) patterns were conducted using a Bruker D8 Advance X-Ray diffractometer with a Cu Kα anode (λ= 0.15406 nm) at 40 kV and 40 mA.

**Thermogravimetric analysis of ZIF-8, Enzyme/ZIF-8 composites**

Samples were heated from room temperature to 600 ^o^C at a rate of 10 oC/min under air atmosphere on a TA Instruments TGA 2050 Thermogravimetric Analyzer.

**Activity assay of enzyme/ZIF-8 composite and its free counterpart**

The activity of HRP was determined by measuring the rate of decomposition of hydrogen peroxide with THB, which can be converted to a yellowish product, purpurogallin, detectable at 420 nm.1 In a typical experiment, HRP/ZIF-8 was added to a solution containing H_2_O_2_ (9 μM) and THB (16 mM) in phosphate buffer saline. After reaction for 10 min, the solution was centrifuged for 2 min at 12000 rpm. And the absorbance of the supernatant was recorded at 420 nm on a UV-Vis spectrophotometer. The activity of free HRP was measured using the same procedure.

For the enzymatic activity determination of Cyt c/ZIF-8 and free Cyt c, similar procedure was followed by shortening the reaction time to 3 min.

For the activity assay of CALB/ZIF-8, p-NPB was first dissolved in acetone and then diluted with phosphate buffer (50 mM, pH 7.0) containing 1.25% (w/v) Triton X-100. The composite of CALB/ZIF-8 was added to the phosphate buffer containing 4-nitrophenyl butyrate (p-NPB) (0.5 mM) to initiate the reaction. After reaction for 3 min, the solution was centrifuged at 12000 rpm for 2 min. The absorbance of the supernatant was determined at 400 nm by using a UV/Vis spectrophotometer.

**Enzyme stability in denaturing organic solvents**

Enzyme/ZIF-8 composites and corresponding free enzyme powders were incubated in water, dimethyl sulfoxide (DMSO), dimethyl formamide (DMF) at 80 ^o^C and in boiling methanol and ethanol for 1 h. Tiny amount of the enzyme solution was taken out and diluted to appropriate concentration and subjected to the above enzymatic assays. The relative activity of enzyme/ZIF-8 was calculated as the ratio of the activity of treated enzyme/ZIF-8 exposing to high temperature and organic solvents after required immersion time and activity of the untreated enzyme/ZIF-8 (Equation 1). The activity of the untreated enzyme/ZIF-8 was set as 100%. The relative activity of free enzyme was calculated in the same way.

$Relative\text{ }Activity=\frac{Activity\text{ }of\text{ }Enzyme/\mathrm{ZIF}-8\text{ treated}}{Activity\text{ }of\text{ }Enzyme/\mathrm{ZIF}-8\text{ untreated}}\times100\%$ (Equation 1)


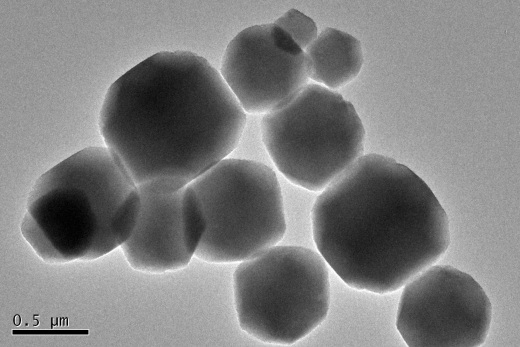


**Figure S1.** TEM image of ZIF-8


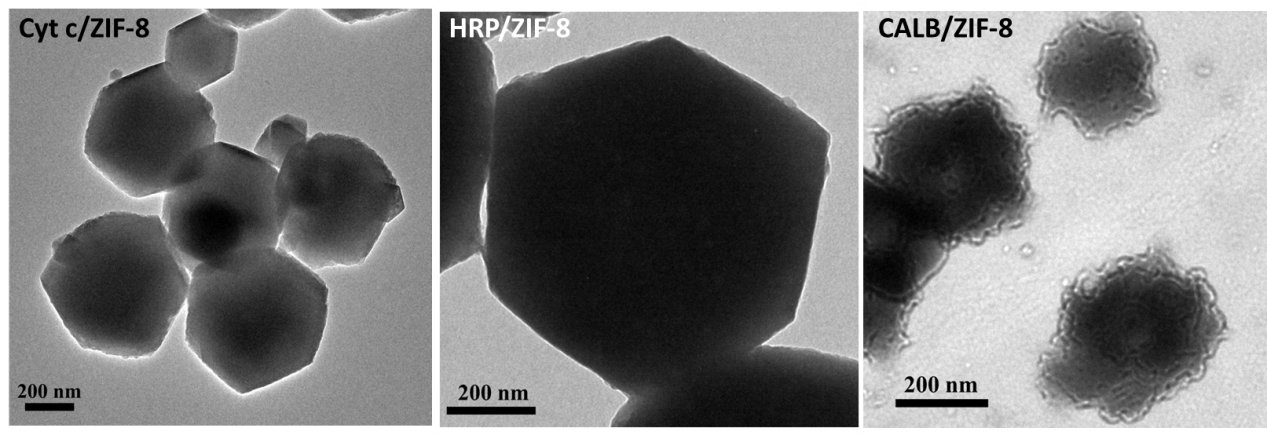


**Figure S2.** TEM images of Cyt c/ZIF-8, HRP/ZIF-8 and CALB/ZIF-8





**Figure S3.** TGA curves of ZIF-8 and enzyme/ZIF-8 composites


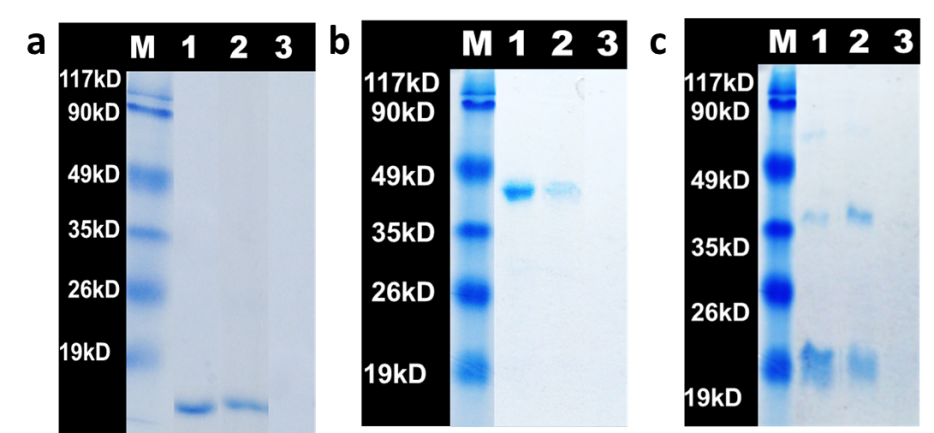


**Figure S4.** SDS-PAGE analysis: (M: protein marker, lane 1: free enzyme, lane 2: washed and digested enzyme/ZIF-8; lane 3: washed and digested enzyme@ZIF-8), Cyt c (a), MW:12 kDa; HRP (b), MW:44 kDa; CALB (c), MW: 38 kDa





**Figure S5.** Storage stability of HRP/ZIF-8 and free HRP





**Figure S6.** Recycling performance of HRP/ZIF-8

**Reference**

1. Q. Wang, Z. Yang, L. Wang, M. Ma and B. Xu, *Chem. Commun.*, 2007, 1032.
